# Supplementary material for: Rapid generation of ventral A9-like dopaminergic neurons from patterned iPSCs
Source: bioRxiv. 2025 Nov 2:2025.10.31.685897. Preprint. [Version 1] doi: 10.1101/2025.10.31.685897 (PMC12636497; doi:10.1101/2025.10.31.685897)

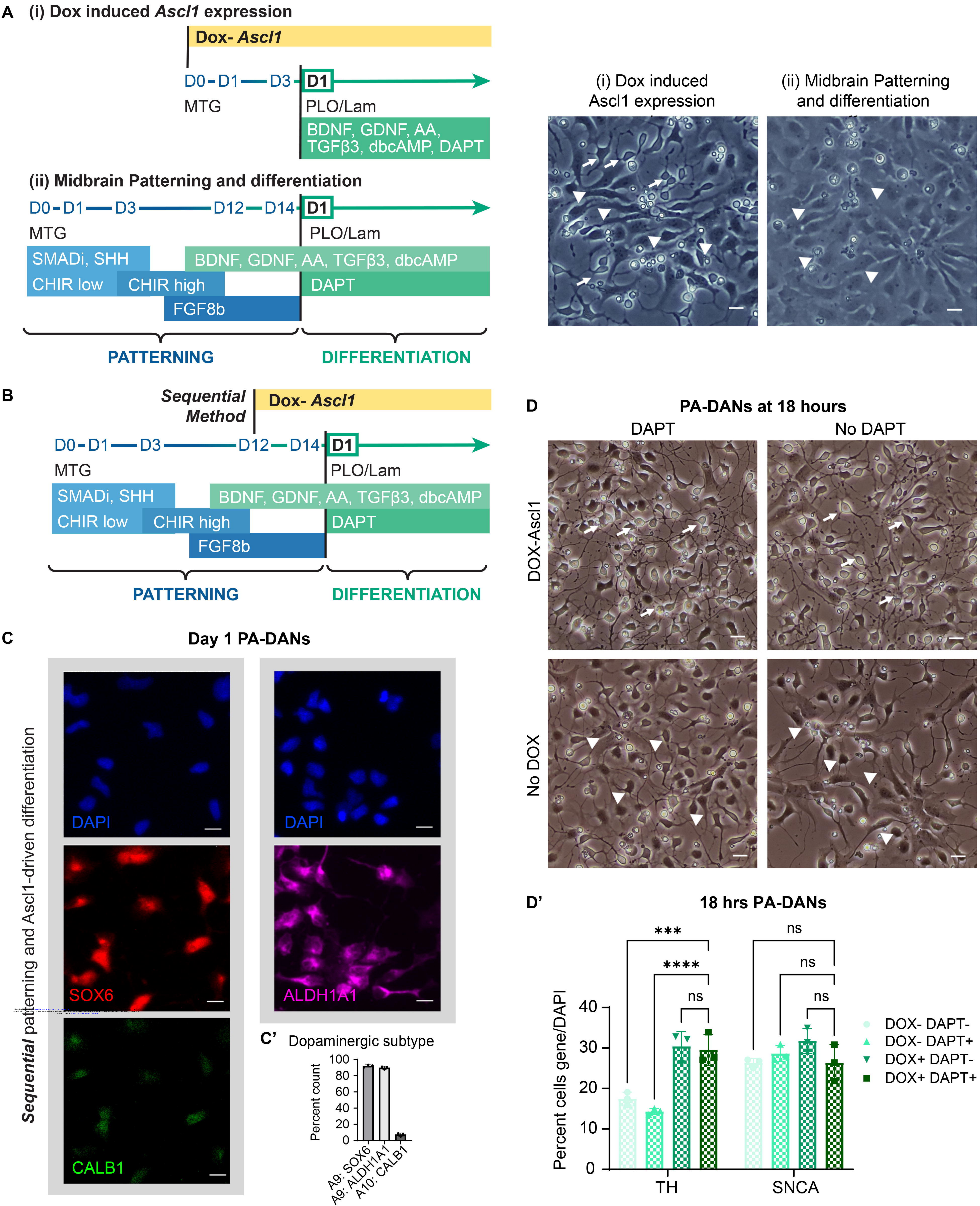
A
(i) Dox induced Ascl1 expression
Dox- Ascl1
D0 D1 D3 D1
MTG
PLO/Lam
BDNF, GDNF, AA, TGFβ3, dbcAMP, DAPT
(ii) Midbrain Patterning and differentiation
D0 D1 D3 D12 D14 D1
MTG
PLO/Lam
SMADi, SHH
CHIR low
CHIR high
FGF8b
BDNF, GDNF, AA, TGFβ3, dbcAMP
DAPT
PATTERNING
DIFFERENTIATION
(i) Dox induced Ascl1 expression
(ii) Midbrain Patterning and differentiation
B
Sequential Method
Dox- Ascl1
D0 D1 D3 D12 D14 D1
MTG
PLO/Lam
SMADi, SHH
CHIR low
CHIR high
FGF8b
BDNF, GDNF, AA, TGFβ3, dbcAMP
DAPT
PATTERNING
DIFFERENTIATION
C
Day 1 PA-DANs
Sequential patterning and Ascl1-driven differentiation
DAPI
SOX6
CALB1
DAPI
ALDH1A1
C'
Dopaminergic subtype
Percent count
0
20
40
60
80
100
A9: SOX6
A9: ALDH1A1
A10: CALB1
D
PA-DANs at 18 hours
DAPT
No DAPT
DOX-Ascl1
No DOX
D'
18 hrs PA-DANs
***
****
ns
ns
ns
ns
Percent cells gene/DAPI
0
10
20
30
40
TH
SNCA
DOX- DAPT-
DOX- DAPT+
DOX+ DAPT-
DOX+ DAPT+

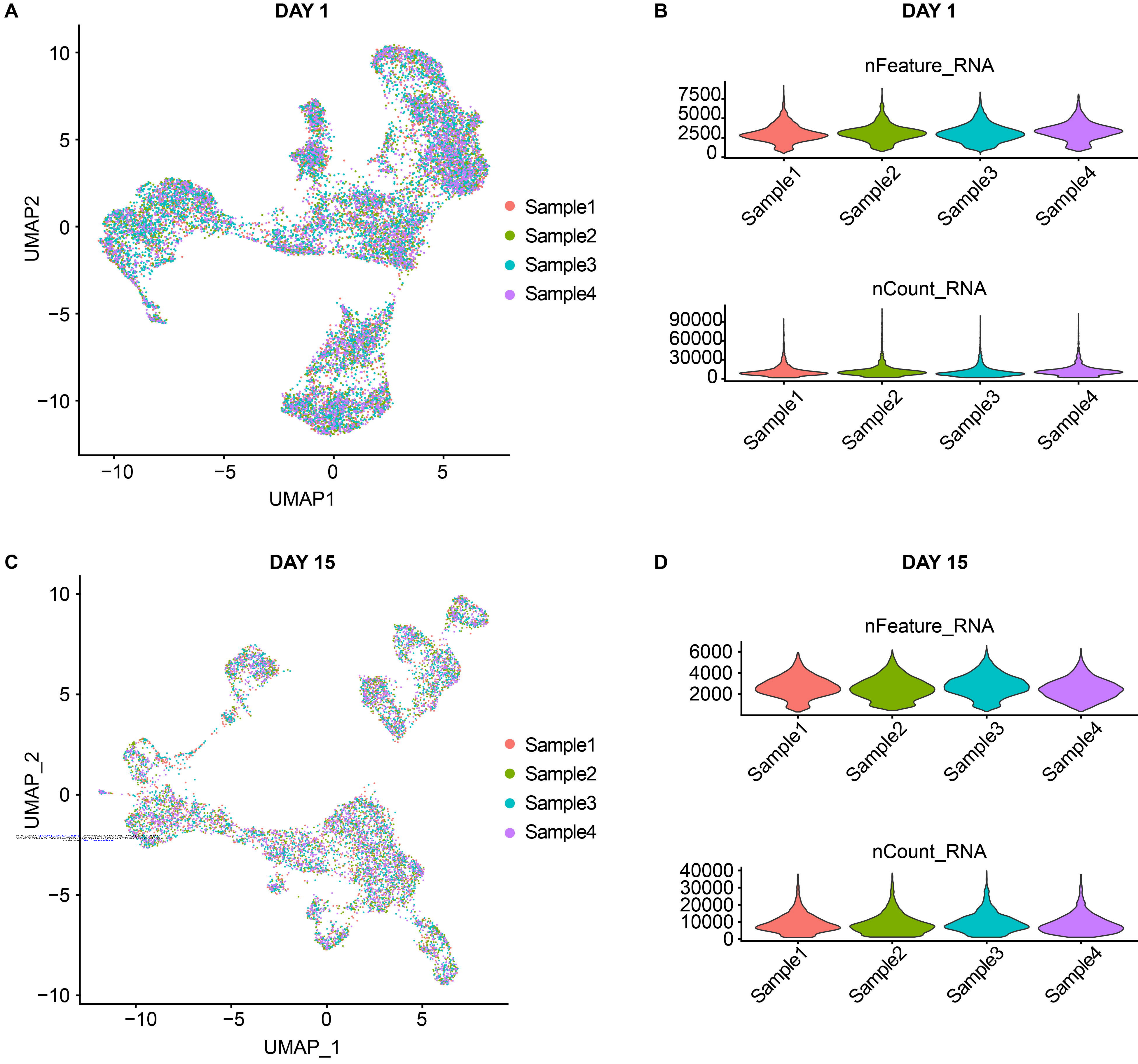


bioRxiv preprint doi: https://doi.org/10.1101/2025.10.31.685897; this version posted November 2, 2025. The copyright holder for this preprint (which was not certified by peer review) is the author/funder, who has granted bioRxiv a license to display the preprint in perpetuity. It is made available under aCC-BY 4.0 International license.

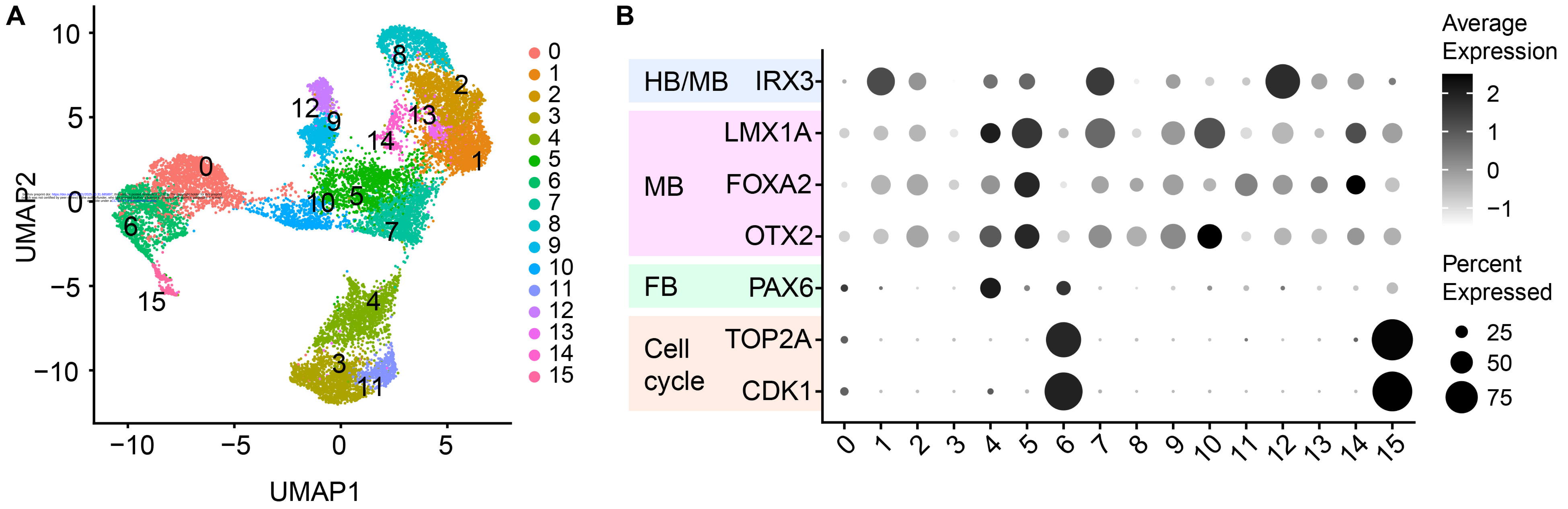


bioRxiv preprint doi: https://doi.org/10.1101/2025.10.31.685897; this version posted November 2, 2025. The copyright holder for this preprint (which was not certified by peer review) is the author/funder, who has granted bioRxiv a license to display the preprint in perpetuity. It is made available under aCC-BY 4.0 International license.

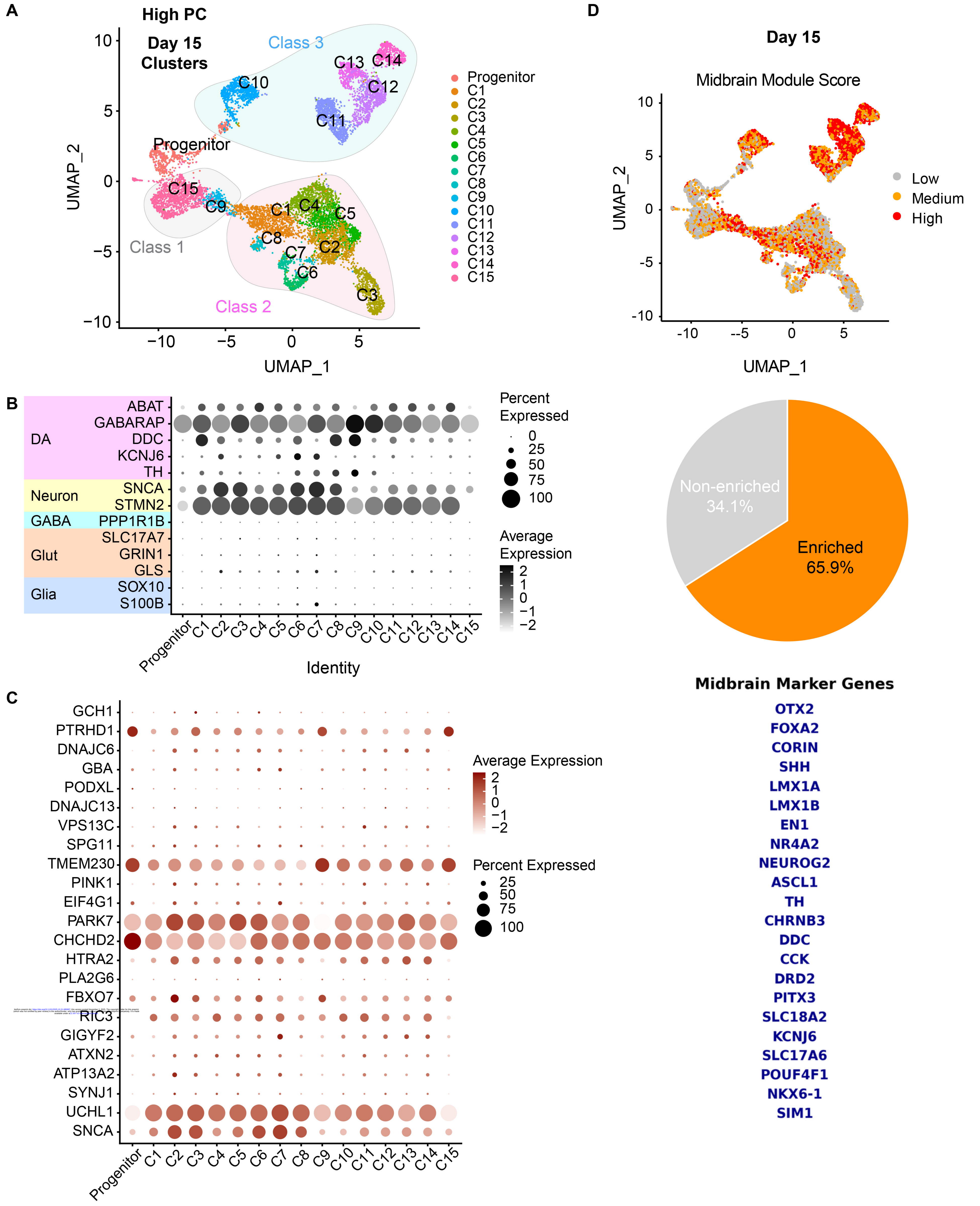
A
High PC
Day 15
Clusters
Class 3
Class 1
Class 2
Progenitor
C1
C2
C3
C4
C5
C6
C7
C8
C9
C10
C11
C12
C13
C14
C15
UMAP_1
UMAP_2
B
DA
Neuron
GABA
Glut
Glia
ABAT
GABARAP
DDC
KCNJ6
TH
SNCA
STMN2
PPP1R1B
SLC17A7
GRIN1
GLS
SOX10
S100B
Identity
Percent Expressed
Average Expression
C
GCH1
PTRHD1
DNAJC6
GBA
PODXL
DNAJC13
VPS13C
SPG11
TMEM230
PINK1
EIF4G1
PARK7
CHCHD2
HTRA2
PLA2G6
FBXO7
RIC3
GIGYF2
ATXN2
ATP13A2
SYNJ1
UCHL1
SNCA
Average Expression
Percent Expressed
D
Day 15
Midbrain Module Score
Low
Medium
High
Non-enriched
34.1%
Enriched
65.9%
Midbrain Marker Genes
OTX2
FOXA2
CORIN
SHH
LMX1A
LMX1B
EN1
NR4A2
NEUROG2
ASCL1
TH
CHRNB3
DDC
CCK
DRD2
PITX3
SLC18A2
KCNJ6
SLC17A6
POUF4F1
NKX6-1
SIM1

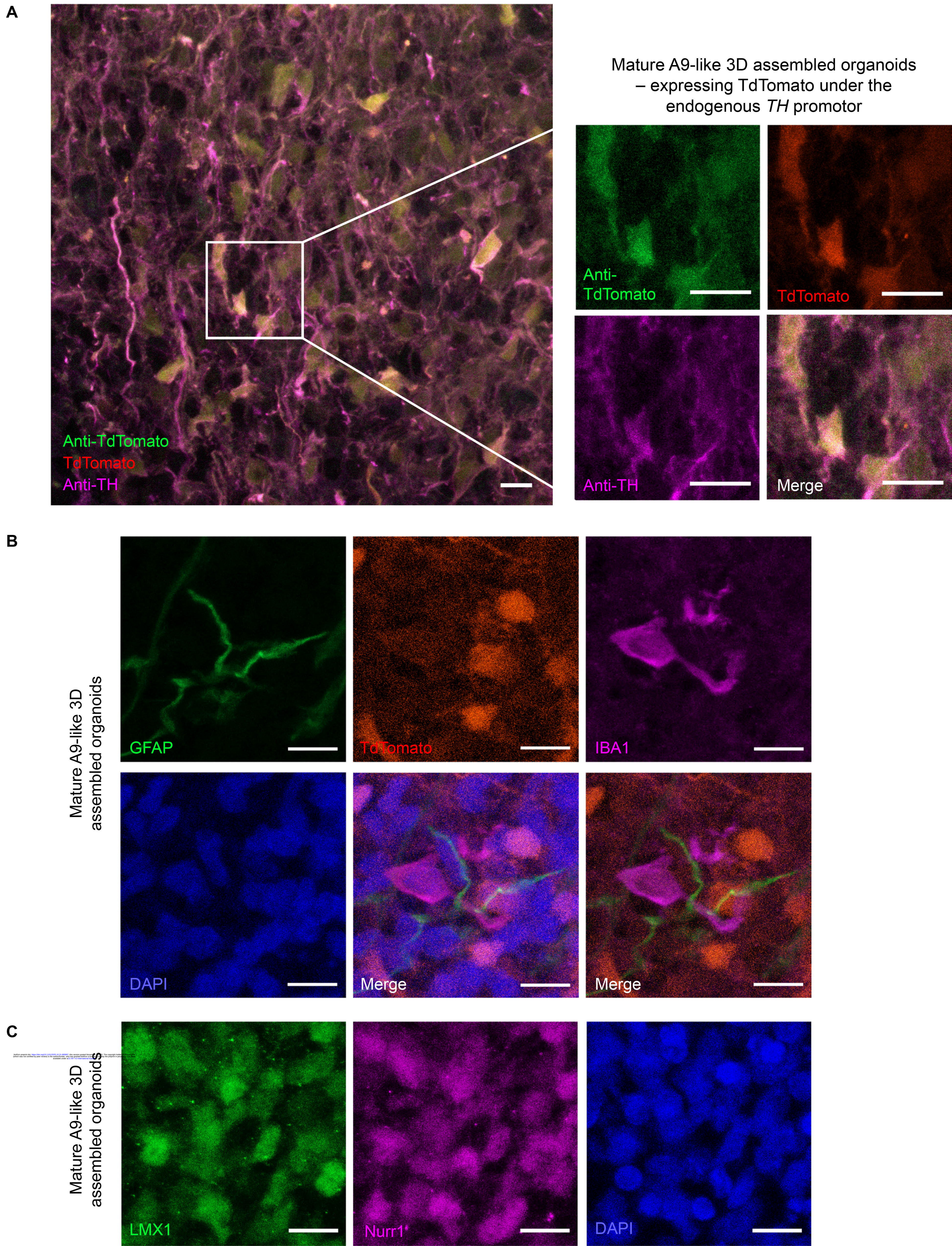
A
Mature A9-like 3D assembled organoids – expressing TdTomato under the endogenous *TH* promotor
Anti-TdTomato
TdTomato
Anti-TH
Anti-TdTomato
TdTomato
Anti-TH
Merge
B
Mature A9-like 3D assembled organoids
GFAP
TdTomato
IBA1
DAPI
Merge
Merge
C
Mature A9-like 3D assembled organoids
LMX1
Nurr1
DAPI

**A** Wildtype KOLF2.1J astrocytes 6 months old

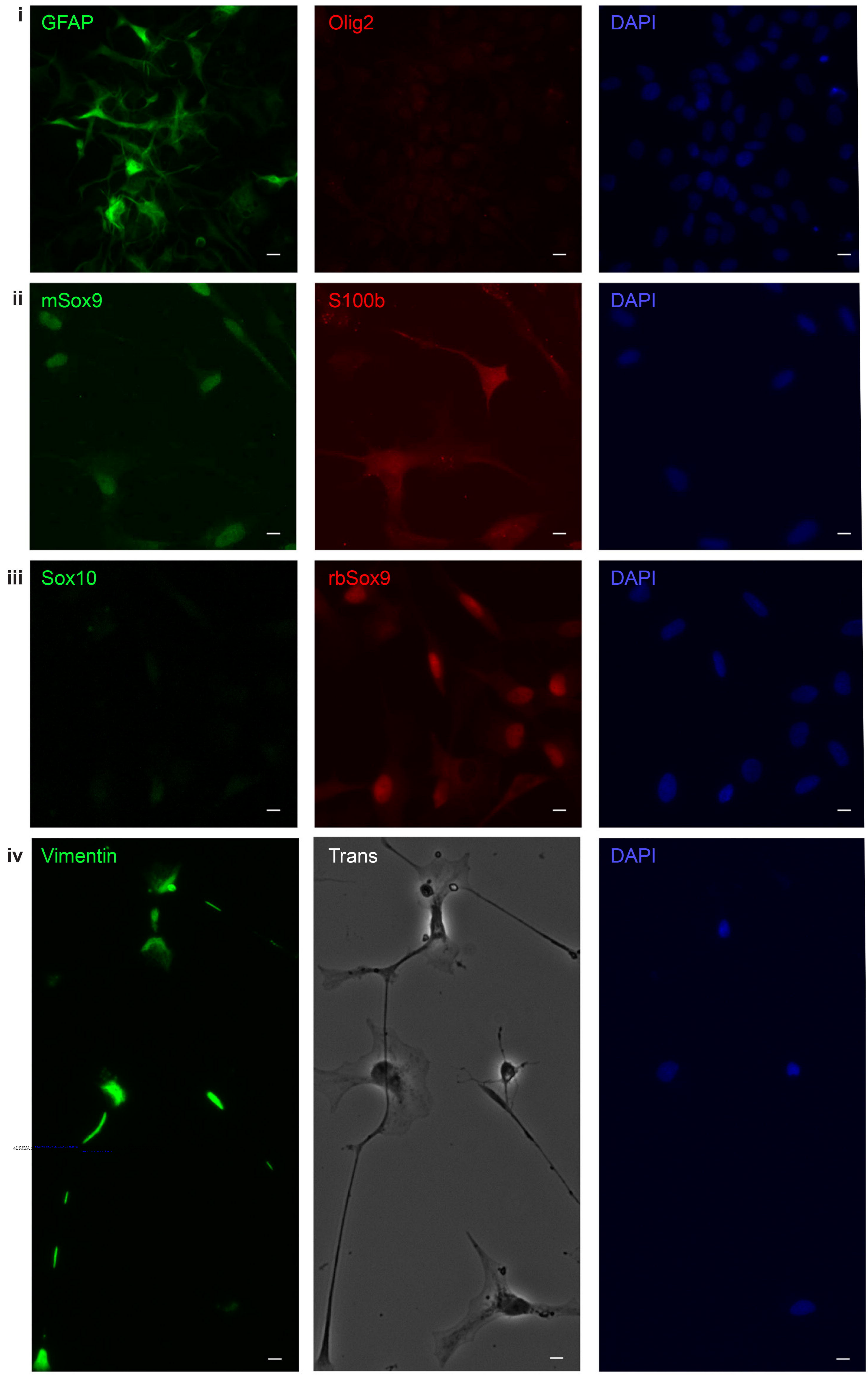

Supplement: Supplement 2 [file NIHPP2025.10.31.685897v1-supplement-2.pdf]
